# Using Text Messages and Fotonovelas to Increase Return of Home-Mailed Colorectal Cancer Screening Tests: Mixed Methods Evaluation

**DOI:** 10.2196/39645

**Published:** 2023-03-07

**Authors:** Carly E Levitz, Elena Kuo, Monica Guo, Esmeralda Ruiz, Evelyn Torres-Ozadali, Rena Brar Prayaga, Anne Escaron

**Affiliations:** 1 Center for Community Health and Evaluation Kaiser Permanente Washington Health Research Institute Seattle, WA United States; 2 Institute for Health Equity AltaMed Los Angeles, CA United States; 3 mPulse Mobile Inc Encino, CA United States

**Keywords:** colorectal cancer screening, texting campaign, patient navigation, fotonovela, fecal immunochemical test kit, FIT kit, screening, cancer, colorectal cancer, CRC, bidirectional texting, health text messaging, health promotion, participation, fotonovela, comics

## Abstract

**Background:**

Colorectal cancer (CRC) is currently the second leading cause of cancer-related deaths in the United States; however, it is mostly preventable with appropriate screening and is often treatable when detected at early stages. Many patients enrolled in an urban Federally Qualified Health Center (FQHC) clinic were found to be past due for CRC screening.

**Objective:**

This study described a quality improvement (QI) project to improve CRC screening rates. This project used bidirectional texting with fotonovela comics and natural language understanding (NLU) to encourage patients to mail fecal immunochemical test (FIT) kits back to the FQHC.

**Methods:**

The FQHC mailed FIT kits to 11,000 unscreened patients in July 2021. Consistent with the usual care, all patients received 2 text messages and a patient navigator call within the first month of mailing. As part of a QI project, 5241 patients who did not return their FIT kit within 3 months, aged 50-75 years, and spoke either English or Spanish were randomized to either usual care (no further intervention) or intervention (4-week texting campaign with a fotonovela comic and remailing kits if requested) groups. The fotonovela was developed to address known barriers to CRC screening. The texting campaign used NLU to respond to patients’ texts. A mixed methods evaluation used data from SMS text messages and electronic medical records to understand the impact of the QI project on CRC screening rates. Open-ended text messages were analyzed for themes, and interviews were completed with a convenience sample of patients to understand barriers to screening and impact of the fotonovela.

**Results:**

Of the 2597 participants, 1026 (39.5%) in the intervention group engaged with bidirectional texting. Participating in bidirectional texting was related to language preference (*χ*^2^_2_=11.0; *P*=.004) and age group (*χ*^2^_2_=19.0; *P*<.001). Of the 1026 participants who engaged bidirectionally, 318 (31%) clicked on the fotonovela. Furthermore, 54% (32/59) of the patients clicked on the fotonovela and responded that they loved it, and 36% (21/59) of patients responded that they liked it. The intervention group was more likely to get screened (487/2597, 18.75%) than those in usual care (308/2644, 11.65%; *P*<.001), and this pattern held, regardless of demographic subgroup (sex, age, screening history, preferred language, and payer type). Interview data (n=16) indicated that the text messages, navigator calls, and fotonovelas were well received and not unduly invasive. Interviewees noted several important barriers to CRC screening and offered suggestions for reducing barriers and increasing screening.

**Conclusions:**

Texting using NLU and fotonovela is valuable in increasing CRC screening as observed by the FIT return rate for patients in the intervention group. There were patterns in which patients did not engage bidirectionally; future work should investigate how to ensure that populations are not left out of screening campaigns.

## Introduction

### Background

Colorectal cancer (CRC) is the second leading cause of cancer-related deaths in the United States, accounting for an estimated 53,200 deaths in 2020 [1]. CRC is mostly preventable with appropriate screening and can be treated successfully (5-year survival rate of approximately 90%) when detected at early stages and the cancer is localized [1]. One screening tool for CRC is the fecal immunochemical test (FIT) kits, which have shown promise in increasing screening rates [2]. A yearly FIT is a recommended screening method for asymptomatic adults aged ≥45 years who are at an average risk of CRC [3]. Findings from a Participatory Research to Advance Colon Cancer Prevention pilot study showed that patients with no prior history of CRC screening are more likely to respond to more intensive communication modalities [4] and that some unscreened populations may require multiple outreach and education modalities and touchpoints [5].

The Federally Qualified Health Center (FQHC) that conducted this project has a majority of patients who are Hispanic or Latin American. Hispanic and Latin American people are less likely to be diagnosed at an early stage than non-Latin White people and more likely to be diagnosed with advanced disease. Barriers to CRC screenings can include health beliefs or cultural linguistic barriers (eg, I feel fine, do not need it, it is embarrassing, and it is unpleasant) [6]. In the state of California, where the FQHC is located, Medi-Cal is the State’s version of Medicaid, a benefit program in the United States that pays for medical services for patients with a low-income status. By serving patients with Medicaid, the FQHC supports increasing access to health care and addressing health equity.

A visual narrative approach using fotonovelas—comics that impart a particular message, or short stories—has been piloted with a wide range of users and is narrowing the health equity gap for Spanish speakers and underserved or marginalized populations [7,8]. However, it has typically been used by programs to increase knowledge about screenings and vaccinations [7-10], rather than to directly increase screening rates.

Texting campaigns have been successfully used for health promotion purposes [11] to motivate behavioral change. However, few studies have addressed the effectiveness of texting in supporting CRC screening and colonoscopy preparation [12]. Some studies have used texting campaigns to send one-way text message reminders and educational content to patients [13-15], but few studies have used bidirectional texting, in which the system is built for patients to reply to the initial text messages and receive automated responses from the texting platform [5,16-18].

### Objectives

The goal of this quality improvement (QI) project was to evaluate the impact of tailored SMS text messaging and fotonovela visual stories on patients who remained unscreened in returning FIT kits after the FQHC’s initial outreach attempts. This project sought to understand the success factors, challenges, barriers, and patient experiences to support program improvement.

Our bidirectional texting plus fotonovela intervention builds on established research as well as our own patient-centered research to understand and address patient barriers to behavioral change [4]. The aim of this paper was to report on texting campaign engagement and CRC screening in the context of patient characteristics in the usual care group compared with the intervention group. The QI project includes (1) bidirectional texting that tailors responses to better address individual barriers and (2) the fotonovela visual component that incorporates learnings about patient barriers to build a compelling story. Additional information about how the fotonovelas were created and how natural language understanding was used can be found in a separate study [19].

## Methods

### Patient Population

The QI project was conducted at an FQHC that served approximately 300,000 patients in a large urban environment in California. Per usual care, the FQHC mailed FIT screening kits (n=11,000) to unscreened patients in July 2021. All patients received a text message before the mailing, including a link to an instructional video on completing the FIT kit (ie, a primer text message), a follow-up text message reminding them to complete the FIT kit (ie, reminder text message), and a call from a patient navigator about receiving and completing their FIT within the first month of mailing if the patient had not yet sent it. 12 weeks after the kits were mailed, approximately 60% of the patients outreached did not return the FIT. The nonresponder group (5241 patients, aged 50-75 years, and who spoke either English or Spanish) was enrolled in the QI project to try a novel approach to increase screening rates. A total of 374 patients were excluded because they did not have a valid mobile phone number in the electronic health records.

### Randomization of Nonresponders

Patients were randomized to either the usual care group (no further intervention beyond usual care) or the intervention group (4-week SMS text messaging campaign with a visual story [also called a comic or fotonovela] and the opportunity to request a replacement FIT kit if needed). The randomization was conducted by mPulse Mobile (a third-party texting service) using a Microsoft Excel (Microsoft Corporation) randomizer function and then verified using 2-tailed *t* tests of the mean values of the usual care group versus the intervention group. Patients were block randomized by binary sex (male or female), age group (50-60 and 61-75 years), and prior screening history (Table 1). Screening history was categorized as never screened (never completed a CRC screening), very inconsistent (previous CRC screening was >24 months ago), or inconsistent (CRC screening occurred 12-24 months prior). Language preference (Spanish or English) was used as an inclusion criterion (excluding members who preferred a different language).

**Table 1:** Demographics of usual care and intervention patients after randomization.

Randomization variables				Usual care (n=2644)		Intervention (n=2597)		*P* value
Female, n (%)				1446 (54.69)		1405 (54.1)		.67
Average age (years), mean (SD)				60 (6.2)		60.2 (6.2)		.30
Aged 50-60 years, n (%)				1494 (56.51)		1479 (56.95)		.74
Aged 61-75 years, n (%)				1150 (43.49)		1118 (43.05)		.74
**CRC^a^ screening history, n (%)**								
	Inconsistent		614 (23.22)		603 (23.2)		.99	
	Very inconsistent		800 (30.26)		787 (30.3)		.97	
	Not screened		1230 (46.52)		1207 (46.5)		.97	
**Other important variables**								
	Population whose preferred language is Spanish, n (%)		1670 (63.16)		1599 (61.6)		.23	
	**Insurance payer, n (%)**							
		Commercial	227 (8.58)		211 (8.1)		.55	
		Medi-Cal	1748 (66.11)		1671 (64.3)		.18	
		Medicare	373 (14.11)		431 (16.6)		.01	
		Nonmanaged care	114 (4.31)		115 (4.4)		.84	
		Uninsured	182 (6.88)		169 (6.5)		.59	
	**SDOH index^b^, n (%)**		2371 (17.8)		2330 (18.5)		.15	
		Very low impact	22 (0.93)		28 (1.2)		.36	
		Low impact	82 (3.46)		91 (3.9)		.42	
		Medium impact	224 (9.45)		240 (10.3)		.33	
		High impact	560 (23.62)		555 (23.8)		.87	
		Very high impact	1483 (62.55)		1416 (60.8)		.21	
		Missing SDOH, n (%)	273 (10.32)		267 (10.28)		.96	

^a^CRC: Colorectal cancer.

^b^A Social Determinants of Health (SDOH) index score (0-100) for each patient was generated, where 0 represents a low-needs census tract and 100 represents a high-needs area. Briefly, 5 SDOH bands were used: very low impact (0-20), low impact (20-40), medium impact (40-60), high impact (60-80), and very high impact (80-100), as well as a group of unknown SDOH impact if addresses were not recognized by the system.

### QI Project to Increase Colon Cancer Screening

**Figure 1:**
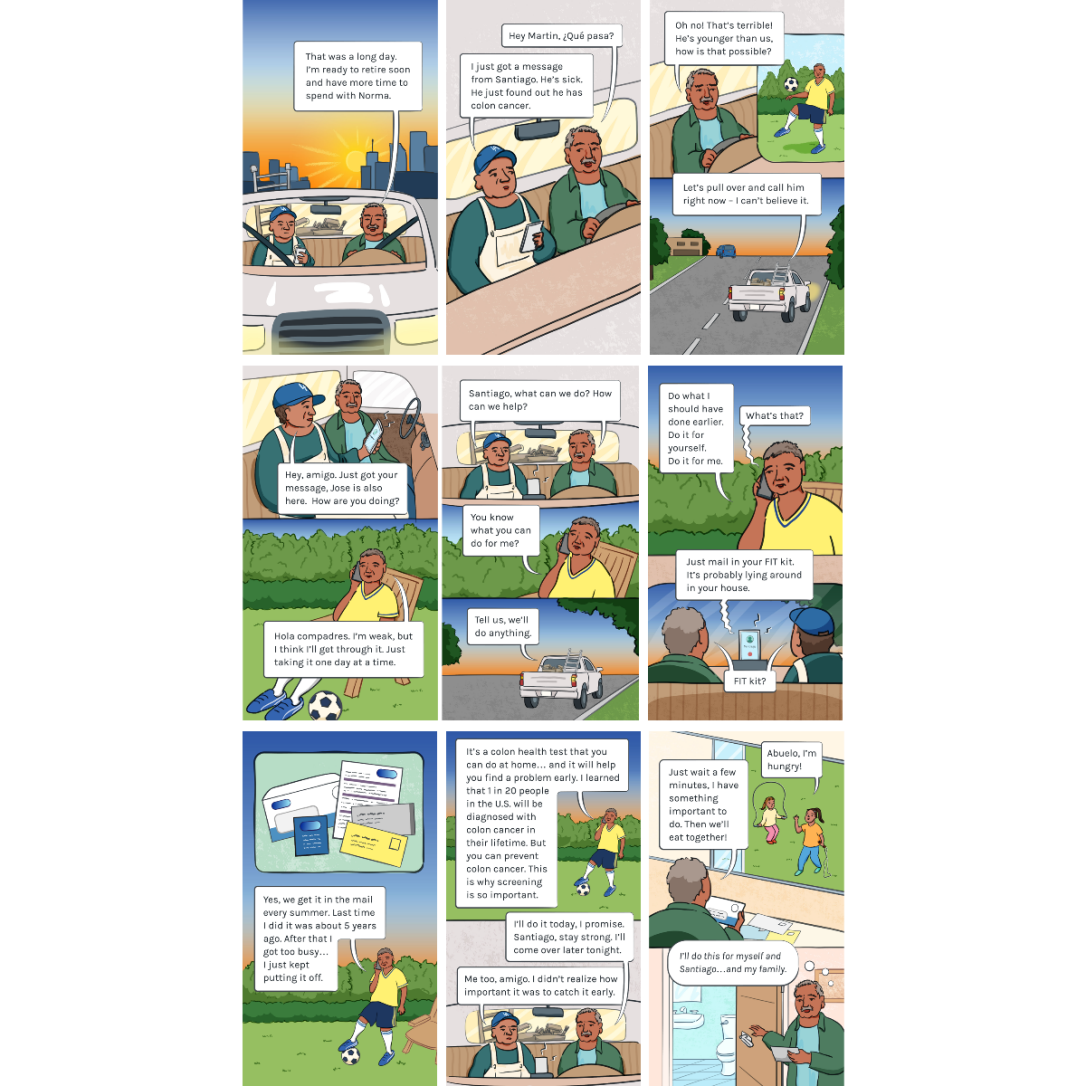
Fotonovela example “Do It for Me” aimed at English-speaking men and English-speaking people of unknown sex.

The 4-week series of text messages was designed and implemented using mPulse Mobile to remind and encourage patients to return their FIT kit. All text messages were in the patient’s preferred language (English or Spanish) at a sixth-grade reading level or lower. If they responded, natural language understanding was used to trigger appropriate automated replies (Multimedia Appendix 1). The series of messages entailed the following:

1. Week 1 was tailored to prior screening history and promoted CRC screening literacy. For those who had never been screened, the message included a comment about “Do it for your peace of mind and your health!” For those that were inconsistent or very inconsistent, the message was modified to say, “We know you’ve completed colon cancer screening before- but you are due now. We’ll check back in about a week.”
2. Week 2 addressed barriers to screening by asking: “If you haven’t done it yet, please tell us if any of these reasons apply” and then followed up with automated conversational responses specific to the barriers the patient reported. The provided reasons included: “1. I’m not sure why I need it”; “2. I feel fine, and I don’t have any pain or symptoms”; “3. I’m too busy right now”; “4. I’m scared about the results”; and “5. It’s embarrassing to do it and then mail it back.” Patients could reply using numeric responses (1-5) or use their own words to share why they had not returned the FIT kit.
3. Week 3 asked patients to click on a link to view a “comic about FIT kits and why you should get it done soon.” Clicking on the link loaded a fotonovela in the mobile browser tailored to their sex and language preferences. Characters within the fotonovela talked about the FIT kit, addressed myths and misconceptions, highlighted the need for self-care and the dangers of procrastination, and emphasized the value of prevention for individuals and their families (Figure 1).
4. Week 4 reminded patients to complete and return the FIT kit, and the patients who replied that they had mailed it in were told what to expect next if their result was normal versus abnormal (ie, blood in stool). Those who had not yet sent it were reminded of the final time: “Do try to get this done as soon as possible. It’s quick and easy, and you will be protecting yourself against colon cancer.”

Many of the automated text messages contained questions with close-ended responses that the patients could text back (Multimedia Appendix 1). In addition, patients could text back in their own words, and those responses were handled using rules and basic natural language processing and monitored using mPulse Mobile. For example, if a patient texted “what is a FIT kit?” or “why do I need a FIT kit?” they received an automated response saying “A FIT is a quick and easy test to find blood in your stool (poop) that you might not be able to see. If you have hidden blood, we ask you to get a colonoscopy. This looks for any growth that we can remove before they turn into colon cancer.” The message also provided the FQHC’s phone number in case they had further questions or required support. Similarly, in instances where patients were familiar with the test but did not believe it was necessary and replied, “I feel fine” or “I don’t need the test,” the intervention built knowledge and health literacy by texting back, “It turns out most people with colon cancer feel healthy and have no symptoms. And most people with colon cancer also have no family history of the disease. This is a quick and easy way to find out if there are any problems.” Again, they were reminded that they could call the FQHC and were provided with the phone number to feel free to ask any questions about why they needed to complete the test.

When patients requested a new FIT kit or replied that they did not receive the FIT kit, an automated text message asked them to request one at their next visit, and mPulse Mobile provided the patients’ information back to the FQHC so they could mail a new FIT kit to them (n=200). If a patient opted out by replying “STOP” or “WRONG” at any point, they received no further text messages. A patient could engage and later decide to opt out. If a patient texted “Help,” then the automated response included the phone number to the FQHC’s patient service center.

In combination with the automated responses via text messages, fotonovelas were created to address barriers found in the literature such as procrastination, lack of self-care, lack of time, embarrassment about the process, and fear of results [20-25]. Fotonovelas were written in both Spanish and English, and each version contained a cast of either men or women for a total of 4 different fotonovelas. They contained a story about someone encouraging a friend to complete their FIT, explaining why it is important to do so, and normalizing the process. The fotonovela comes with a call to action for patients to use the kit that they received in the mail (Figure 1).

### Quantitative Data Analysis and Data Sources

There were 2 data sources: one from the FQHC based on electronic medical record data and one from mPulse Mobile. The data from the 2 sources were linked using a unique identifier common in both data sets. The data were transferred via secure file transfer options. All quantitative data from texting outreach and electronic medical records were analyzed using R (R Foundation for Statistical Computing). The *t* tests of the mean values of screening completion rates were used to test the difference between the intervention and usual care groups, as well as to verify the distribution of demographic characteristics. Differences in texting engagement and clicking on the fotonovela by population characteristics were tested using chi-square statistics for categorical variables. Logistic regressions were run for clicking the fotonovela (no=0, yes=1) and for being screened (no=0, yes=1) to consider covariates that could be related to each of these 2 key outcomes. Differences in FIT results (normal, abnormal, erroneous [ie, FIT needs to be repeated, or no FIT returned]) in the 2 groups (usual care vs intervention) were tested using chi-square statistics for categorical variables.

### CRC Screening Completion

CRC screening completion and results were determined by running a report querying the electronic medical records 2 months after the intervention to capture completion based on CRC screening performed by the patient (eg, colonoscopy, FIT). If a patient’s record was updated to indicate that a colonoscopy or another screening method had been performed within the appropriate time frame, they were considered screened. Blood in the stool sample indicated an abnormal result for the FIT kit.

### Covariates

Demographic variables of interest (sex, age, prior screening history, and language preference) were collected from the electronic medical records. Additional variables of interest included insurance payer (commercial, Medi-Cal, Medicare, nonmanaged care, and uninsured), and the Social Determinants of Health (SDOH) index.

The SDOH index was derived from 10 Census-datapoint factors such as unemployment and percent of the population who completed high school (range 0-100, where 0 represents low-needs census tract and 100 represents high-needs area). The index was developed by mPulse Mobile [26] and was used to create 5 bands of need: very low impact, low impact, medium impact, high impact, and very high impact. It provides a granular view of the population at the United States census tract level and can be used to highlight neighborhoods where there might be a higher incidence of unmet social needs and an increased likelihood of health inequities. The SDOH index was included to monitor whether disparities were being mitigated or worsened.

### Engagement in Bidirectional Texting and Fotonovela

mPulse Mobile tracked 2 engagement process measures: whether a patient replied to a text message and whether a patient clicked on the fotonovela. Patients who responded to at least one text message (ie, participated in a bidirectional text exchange) were considered “engaged.” If they responded, but at some point opted out, they were considered “engaged but opted out.” Patients who did not respond to any text message were considered “not engaged.” It was not possible to track whether the patient viewed the fotonovela, only whether they clicked the text message link to the fotonovela (yes or no).

### Barriers to Screening and Impact of Fotonovela

The data were collected both through the texting program as well as by interviews. Through the texting program, patients were asked whether they received the FIT kit in the mail (yes or no). Patients received a text message asking what they thought about the fotonovela and were given the options of “didn’t like it,” “it was okay,” “liked it,” and “loved it.” They were also asked whether the fotonovela would affect their behavior regarding screening in the coming week. Patients’ free-text responses via text message were reviewed to determine whether they completed the FIT, the barriers they experienced in completing the FIT, enjoyment of the fotonovela, and whether the fotonovela would affect their behavior. When possible, open-ended responses were recoded to fit into one of the options provided. Responses that did not fit into the options provided were reviewed for themes, which were analyzed alongside the interview themes.

In addition, phone interviews were conducted to gather feedback on the QI project. A convenience sample of 144 patients was selected to be outreached. The numbers were split evenly among English speakers and Spanish speakers, and there were 4 groups within each language group: patients receiving usual care who completed the FIT, patients receiving usual care who did not complete the FIT, patients receiving a bidirectional automated texting campaign who did not complete the FIT, and patients receiving a bidirectional automated texting campaign who completed the FIT. Potential interviewees were sent text messages up to 3 times, with an invitation to participate in a phone interview. Of the 144 patients, 119 (82.6%) did not respond to the text invitations and 2 (1.4%) declined to participate. A total of 16 patients were interviewed, and an additional 6 patients were scheduled but did not complete the interview. Interview questions were regarding barriers, facilitators, and motivators for completing the FIT kit. Participants in the intervention group were also asked about their perceptions of the fotonovela and what role it played in deciding whether to complete the FIT. Data regarding user experience were themed using emergent coding methods [27].

### Ethics Approval

The QI project was reviewed and determined to not involve research and therefore was exempted by the Kaiser Permanente Washington Human Subjects Review Office. Patients who agreed to be interviewed as part of the QI project received a US $25 Amazon, Starbucks, or Target gift card incentive (patients chose which gift card they would like).

## Results

### Randomization

The *t* tests found no statistical difference between the usual care and intervention groups in the following variables: binary sex, age group, and prior CRC screening history (Table 1). In addition, the intervention and usual care groups had similar distributions of payer types, Spanish language preference, and SDOH index distribution even though they were not randomization variables. The percent of patients receiving Medicare differed between the usual care (373/2644, 14.11%) and intervention (431/2597, 16.6%; *P*=.01) groups. Across both the intervention and usual care groups, >40% had never been screened for CRC. Approximately two-thirds of the patients in each group had Medi-Cal insurance (1748/2644, 66.11% in usual care and 1671/2597, 64.34% in intervention).

### Engagement Through Bidirectional Texting

**Figure 2:**
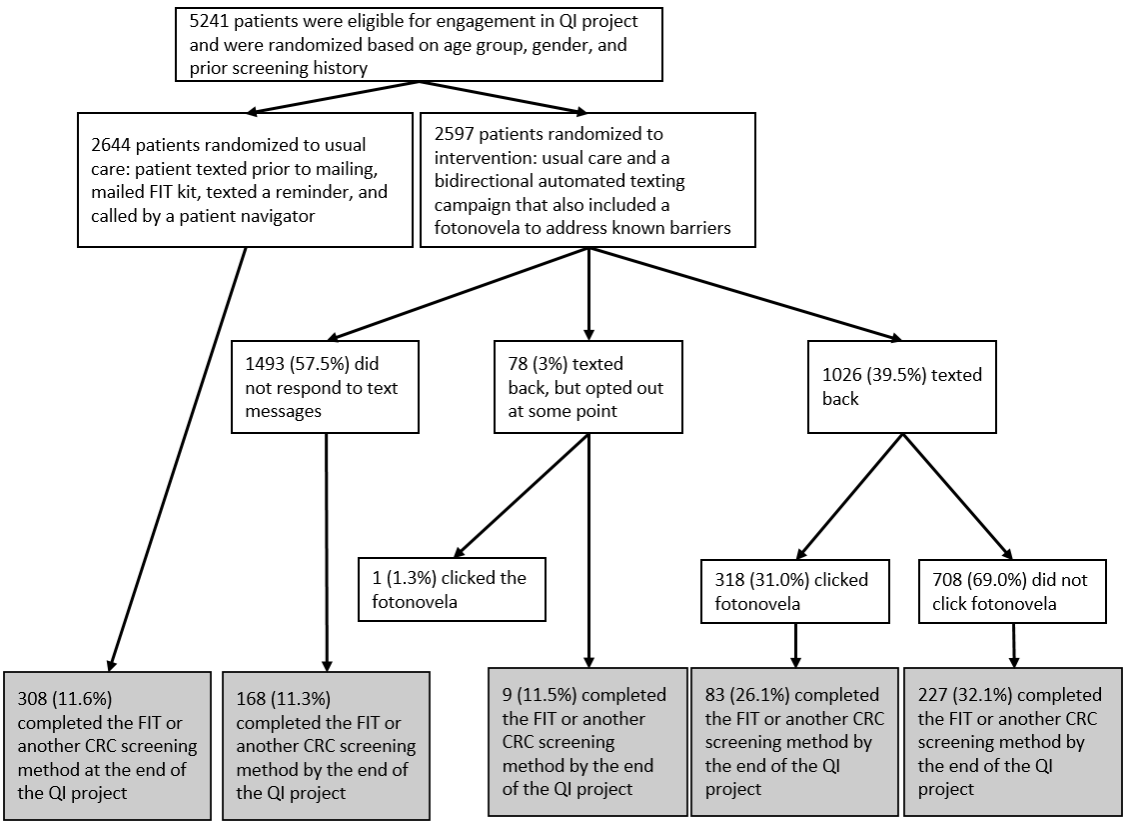
Engagement of patients in the intervention. CRC: colorectal cancer; FIT: fecal immunochemical test; QI: quality improvement.

Approximately 39.51% (1026/2597) of the patients in the intervention group engaged in bidirectional texting. More than half (1493/2597, 57.49%) of the patients in the intervention group did not engage in text messages and 3% (78/2597) texted back “STOP” or “WRONG” and opted out (1 patient opted out after engaging; Figure 2).

Engagement was statistically related to language preference (n=2597; *χ*^2^_2_=11.0; *P*=.004); age group (n=2597; *χ*^2^_2_=19.0; *P*<.001); prior screening history (n=2597; *χ*^2^_4_=14.8; *P*=.005); insurance type (a greater proportion of those who engaged had commercial insurance than those who did not engage or engage but opted out; n=2597; *χ*^2^_8_=27.4; *P*<.001); and SDOH index band, where those who engaged had a higher SDOH index score (n=2330; *χ*^2^_8_=20.4; *P*=.009; Table 2).

**Table 2:** Demographics of patients by engagement category.

Variable			Did not engage (n=1493)		Engaged, but opted out (n=78)		Engaged via text message (n=1026)		Chi-square tests of association (*df*; n=2597)		*P* value
Sex (binary), n (%)			783 (52.44)		42 (53.85)		580 (56.53)		4.1 (2)		.13
Age (years), mean (SD)			60.7 (6.4)		60.7 (6.3)		59.3 (5.8)		19 (2)		<.001
**CRC^a^ screening history, n (%)**								14.8 (4)		.005	
	Inconsistent	316 (21.16)		12 (15.38)		275 (26.8)					
	Very inconsistent	466 (31.21)		22 (28.21)		299 (29.14)					
	Never screened	711 (47.62)		44 (56.41)		452 (44.05)					
Spanish as preferred language, n (%)			927 (62.09)		34 (43.59)		638 (62.18)		11.0 (4)		.004
**Insurance payer, n (%)**								27.4 (8)		<.001	
	Commercial	95 (6.36)		5 (6.41)		111 (10.82)					
	Medi-Cal	969 (64.9)		44 (56.41)		658 (64.13)					
	Medicare	265 (17.75)		15 (19.23)		151 (14.72)					
	Nonmanaged care	70 (4.69)		3 (3.85)		42 (4.09)					
	Uninsured	94 (6.3)		11 (14.10)		64 (6.24)					
**SDOH index band^b^ (n=2330), n (%)**								20.4 (8)		<.009	
	Average SDOH index	1343 (80.3)		70 (73.9)		917 (78.5)					
	Very low impact	12 (0.89)		2 (2.86)		14 (1.5)					
	Low impact	50 (3.72)		6 (8.57)		35 (3.8)					
	Medium impact	121 (9.01)		13 (18.57)		106 (11.6)					
	High impact	313 (23.31)		13 (18.57)		229 (25)					
	Very high impact	847 (63.07)		36 (51.43)		533 (58.1)					
	Missing SDOH, n (%)		150 (10.05)		8 (10.26)		109 (10.6)		20.4 (8)		.009

^a^CRC: colorectal cancer.

^b^An Social Determinants of Health (SDOH) index score (0-100) for each *patient* was generated, where 0 represents a low-needs census tract and 100 represents a high-needs area. Briefly, 5 SDOH bands were used: very low impact (0-20), low impact (20-40), medium impact (40-60), high impact (60-80), and very high impact (80-100), as well as a group of unknown SDOH impacts if addresses were not recognized by the system.

### Engagement Through Clicking Fotonovela Link

Of those who engaged in the bidirectional texting, just less than one-third (319/1026, 31.09%) clicked on the fotonovela link (Figure 2). All but one of the 319 patients who clicked on the fotonovela participated in bidirectional texting without opting out.

For those who engaged in bidirectional texting, there was no association between clicking on the fotonovela and the following variables: binary sex, preferred language, prior CRC screening history, or the SDOH index band (Table 3). Those aged 61-75 years were less likely to click on the fotonovela than those aged 50-60 years (odds ratio=0.67; *P*=.02). Those who did not reply to a text message asking whether they received the FIT kit in the mail were more likely to click on the fotonovela than those who texted “yes” that they did receive the FIT kit in the mail (odds ratio=2.08; *P*<.001). Those with Medicare were more likely to click on the fotonovela than those with commercial insurance (odds ratio=1.91; *P*=.04). Those who engaged but opted out were much less likely to click on the fotonovela than those who engaged (odds ratio=0.02; *P*<.001). These results were consistent with the chi-square analyses for the categorical variables (data not shown).

**Table 3:** Logistic regression predicting whether patients click on the fotonovela in the text message among the patients who engaged via bidirectional texting.

Characteristics		Estimate (SE)	*z* value	*P*>|z|	Odds ratio (95% CI)
Intercept (reference)		−1.31061 (0.3671)	−3.57	<.001	0.27 (0.13-0.55)
Male (reference: female)		0.19289 (0.14868)	1.297	.19	1.21 (0.91-1.62)
61-75 years age band (reference: 50-60)		−0.40128 (0.17102)	−2.346	.02	0.67 (0.48-0.93)
**Screening history (reference: inconsistent)**					
	Never screened	−0.01094 (0.19379)	−0.056	.95	0.99 (0.68-1.45)
	Very inconsistent	0.13551 (0.19741)	0.686	.49	1.15 (0.78-1.69)
Spanish as preferred language (reference: English)		−0.29278 (0.15853)	−1.847	.06	0.75 (0.55-1.02)
**Self-reported receiving FIT^a^** **kit in mail (reference: no)**					
	Unknown	0.73073 (0.20698)	3.531	<.001	2.08 (1.40-3.15)
	Yes	−0.38366 (0.27363)	−1.402	.16	0.68 (0.40-1.16)
**Payer type (reference: commercial)**					
	Medi-Cal	0.32849 (0.26085)	1.259	.21	1.39 (0.84-2.35)
	Medicare	0.66074 (0.32341)	2.043	.04	1.94 (1.03-3.68)
	Nonmanaged care	0.03194 (0.48414)	0.066	.95	1.03 (0.39-2.62)
	Uninsured	0.45208 (0.40198)	1.125	.26	1.57 (0.71-3.45)
**SDOH^b^ band (reference: high impact, 60-80)**					
	Very low impact (0-20)	0.075 (0.57664)	0.13	.90	1.08 (0.33-3.32)
	Low impact (20-40)	−0.47331 (0.43174)	−1.096	.27	0.62 (0.26-1.41)
	Medium impact (40-60)	0.06829 (0.2553)	0.268	.79	1.07 (0.65-1.76)
	Very high impact (80-100)	−0.02755 (0.17733)	−0.155	.88	0.97 (0.69-1.38)
Engaged, but opted out (reference: engaged via bidirectional texting)		−3.77899 (1.01261)	−3.732	<.001	0.02 (0.00-0.11)

^a^FIT: fecal immunochemical test.

^b^SDOH: Social Determinants of Health.

### Patient-Reported Impact of Fotonovela

During the fourth week of the SMS text messaging campaign, 20.7% (66/319) of the patients who participated in bidirectional texting and clicked on the fotonovela responded to a text message query regarding their enjoyment. Of the 59 people who gave a specific rating, 32 (54%) said they loved it, 21 (36%) said they liked it, 6 (10%) said that it was okay, and none said that they did not like it. There were 7 other comments to the text asking the patient to rate the fotonovela, 3 of which were requesting another FIT kit and 1 that was someone saying they were getting a follow-up colonoscopy. Furthermore, 44% (29/66) said that they were more likely to complete the FIT kit after seeing the fotonovela (37 people said that it would not affect their behavior).

Of the 10 interviewees who received the fotonovela, 6 (60%) recalled receiving it and 4 (40%) of them indicated it was helpful (Table 4).

**Table 4:** Barriers to, success factors of, and suggestions for increasing screening from 16 patient interviews.

Category and theme (n=16)	Illustrative quotes from interviews	Patient suggestions for improvement
Barrier: I kept forgetting to do it or did not have it in the bathroom (n=9)	“I kept forgetting until I was already in the bathroom.”	Add a note to place in the bathroom upon receipt.
Barrier: I was busy and did not prioritize it (n=7)	“It takes time to do, and I don’t want to take the extra 10 or 15 minutes to figure out what to do and how to mail or whatever.”	Add an incentive if returned within X number of days, such as a US $5 gift card or entry into a raffle
Barrier: I lost it or did not remember receiving it (n=5)	“My husband picks up the mail, and I don’t know where he put it, but I requested another one when they ([navigator] called, and did it then.”	Include a text with a link to request another kit.
Barrier: It can be difficult or stressful, especially for first time users (n=6)	“The first time I had no idea what to do. They used generic words like ‘open the bag’ what bag? It’s many pieces and lot to read.”	Offer a walk-through at clinic visits; text an offer to request a navigator call if needed, particularly for those who have never completed one.
Barrier: I felt embarrassed to do it (n=2)	“Smearing poop on paper is just weird.”	Acknowledge awkwardness; make it clear that you do not touch fecal material.
Barrier: Did not realize they needed to do it every year (n=2)	“I did it last year and it was negative, so I thought I was good to go, I didn’t know it was an every year thing.”	Include the word “annual” to make the desired frequency clearer.
Success factor: Having the kit mailed to do at one’s convenience in the home (n=15)	“I really like getting mailed kits; it’s much better than doing it at the clinic. I like having the time to sit and read and do it on my own with privacy for something like this.”	Mail kits every year the same month—make it a routine part of care at this clinic.
Success factor: Phone call from patient navigator (n=10)	“Keep having someone call us because that always makes me feel guilty and then I’ll do it. A text I can ignore more easily.”	Add an additional call, especially if another kit is mailed out.
Success factor: Clear instructions with pictures (n=9)	“I didn’t quite get what to do, and the instructions were long and overwhelming. Could you do them in Spanish?”	Consider a video or more pictures, less generic language; keep in mind those who do not read English well.
Success factor: Text Reminders (n=6 out of 10 in intervention group)	“It’s nice to get the text reminder because then the message is there to see when you have time, even if you are busy when it comes in.”	Keep sending text reminders as-is; add texts offering to mail another KIT and texts offering phone support.
Success factor: Fotonovela (n=4 out of 6 who received it)	“The fotonovela made me reflect that I shouldn’t wait, I should not be even more late in doing it!”	Text 1 panel to pique interest and make people more likely to click on the link
Success factor: Family members reminder (n=3)	“My spouse kept bugging me to do it. I know it’s important but it’s just not something you think about doing, I kept putting it off.”	Continue to highlight family in materials—this is something that patients value.

### CRC Screening Completion

If patients returned the FIT kit or underwent colonoscopy, they were considered successfully screened. Patients in the intervention group were significantly more likely to be screened (18.8% screened) compared with those in the usual care group (11.6%; 95% CI for the difference between means was 5.2%-9.0%; *P*<.001; Table 5). This pattern was observed in all demographic subgroups (Table 5). For those who returned the FIT kit, the usual care group had 5.1% (23/448) abnormal results, whereas the intervention group had 2.9% (18/617) abnormal results. FIT results were statistically related to group (N=5241; *χ*^2^_3_=43.3; *P*<.001).

**Table 5:** Screening rates at end of the quality improvement (QI) project for usual care and intervention groups by subgroup.

Percent screened at end of QI project by subgroup		Usual care (n=2644), n (%)	Intervention (n=2597), n (%)	Difference of means (95% CI)	*P* value
*Overall*		308 (11.6)	487 (18.8)	5.2 to 9.0	<.001
**Sex**					
	Male (n=2390)	1198 (10.7)	1192 (16)	2.6 to 8.1	<.001
	Female (n=2851)	1446 (12.4)	1405 (21.1)	5.9 to 11.3	<.001
**Age groups (years)**					
	50-60 (n=2973)	1494 (9.3)	1479 (17.7)	6 to 10.9	<.001
	61-75 (n=2268)	1150 (14.7)	1118 (20.1)	2.3 to 8.5	<.001
**CRC^a^ screening history**					
	Inconsistent (n=1217)	614 (20.8)	603 (32.3)	6.6 to 16.4	<.001
	Very inconsistent (n=1587)	800 (9.9)	787 (15.4)	2.2 to 8.8	<.001
	Never screened (n=2437)	1230 (8.2)	1207 (14.2)	3.5 to 8.5	<.001
**Preferred language**					
	English (n=1972)	974 (7.4)	998 (13.1)	3.1 to 8.4	<.001
	Spanish (n=3269)	1670 (14.1)	1599 (22.3)	5.5 to 10.8	<.001
**Payer type**					
	Commercial (n=438)	227 (15.9)	211 (27.5)	3.9 to 19.3	.003
	Medi-Cal (n=3419)	1748 (10.2)	1671 (16.5)	4 to 8.5	<.001
	Medicare (n=804)	373 (19.6)	431 (26)	0.6 to 12.2	.03
	Nonmanaged care (n=229)	114 (9.6)	115 (15.7)	−2.7 to 14.7	.17
	Uninsured (n=351)	182 (5.5)	169 (14.2)	2.4 to 15	.007
**SDOH^b^ index**					
	Very low impact (n=590)	295 (9.1)	295 (7.1)	−18.1 to 14.2	.81
	Low impact (n=713)	355 (7.3)	358 (11)	−5 to 12.3	.40
	Medium impact (n=1004)	497 (11.2)	507 (17.9)	0.4 to 13.2	.04
	High impact (n=1655)	833 (10.4)	822 (18.9)	4.4 to 12.7	<.001
	Very high impact (n=3439)	1756 (12.4)	1683 (19.6)	4.6 to 9.9	<.001

^a^CRC: colorectal cancer.

^b^SDOH: Social Determinants of Health.

There were large differences in screening rates by demographic variables of interest that were consistent for both intervention and usual care groups (Table 5; logistic regression for the intervention group is provided in Table 6). Men were less likely than women to be screened at the end of the QI project (odds ratio=0.73; *P*=.008). Those with no screening history were less likely to be screened than those with an inconsistent screening history (odds ratio=0.39; *P*<.001). Those with a very inconsistent screening history were also less likely to be screened than those with an inconsistent screening history (odds ratio=0.43; *P*<.001). Those who preferred to speak Spanish were more likely to be screened than those who preferred to speak English (odds ratio=1.75; *P*<.001). Those who self-reported having received the FIT kit in the mail were more likely to be screened than those who self-reported not receiving the FIT kit in the mail (odds ratio=2.85; *P*<.001). Those who engaged (ie, texted bidirectionally) were more likely to be screened than those who did not (odds ratio=3.07; *P*<.001).

**Table 6:** Logistic regression predicting whether patients will be screened at the end of the QI project among the intervention group (n=2597).

Characteristics			Estimate (SE)		*z* value		*P*>|*z*|		Odds ratio (95% CI)
Intercept (reference)			−1.88102 (0.3954)		−4.757		<.001		0.15 (0.07-0.33)
Male (reference: female)			−0.31112 (0.11705)		−2.658		.008		0.73 (0.58-0.92)
61-75 years age band (reference: 50-60)			−0.04095 (0.13063)		−0.313		.75		0.96 (0.74-1.24)
**Screening history (reference: inconsistent)**									
	Never screened	−0.94208 (0.14654)		−6.429		<.001		0.39 (0.29-0.52)	
	Very inconsistent	−0.84281 (0.1467)		−5.745		<.001		0.43 (0.32-0.57)	
Spanish as preferred language (reference: English)			0.55926 (0.13444)		4.16		<.001		1.75 (1.35-2.28)
**Self-reported receiving FIT^a^ kit in mail (reference: no)**									
	Unknown	0.20309 (0.21754)		0.934		.35		1.23 (0.81-1.89)	
	Yes	1.0462 (0.24531)		4.265		<.001		2.85 (1.77-4.64)	
**Engagement (reference: did not engage)**									
	Engaged	1.12186 (0.1485)		7.554		<.001		3.07 (2.29-4.11)	
	Engaged, but opted out	−0.0411 (0.41879)		−0.098		.92		0.96 (0.39-2.05)	
“No” to “clicked fotonovela” (reference: “yes”)			0.1012 (0.17063)		0.593		.55		1.11 (0.79-1.55)
**Payer type (reference: commercial)**									
	Medi-Cal	−0.26009 (0.19647)		−1.324		.19		0.77 (0.53-1.14)	
	Medicare	0.31875 (0.23302)		1.368		.17		1.38 (0.87-2.18)	
	Nonmanaged care	0.05858 (0.35283)		0.166		.87		1.06 (0.52-2.09)	
	Uninsured	−0.07765 (0.32092)		−0.242		.81		0.93 (0.49-1.72)	
**SDOH^b^ band (reference: high impact, 60-80)**									
	Very low impact (0-20)	−0.6958 (0.77029)		−0.903		.37		0.50 (0.08-1.83)	
	Low impact (20-40)	−0.29288 (0.37916)		−0.772		.44		0.75 (0.08-1.83)	
	Medium impact (40-60)	0.08399 (0.21889)		0.384		.70		1.09 (0.70-1.66)	
	Very high impact (80-100)	0.0275 (0.13852)		0.199		.84		1.03 (0.79-1.35)	

^a^FIT: fecal immunochemical test.

^b^SDOH: Social Determinants of Health.

Patients in the intervention group who did not engage (95% CI for difference between means was –2.4% to 1.6%; *P*=.70) or opted out had very similar rates of screening compared with the usual care group (95% CI for difference between means was −7.5% to 7.2%; *P*=.97; Figure 2). Patients in the intervention group who bidirectionally engaged had greater screening rates than those who engaged but opted out (95% CI for difference between means was 10.9%-26.4%; *P*<.001) or than those who did not (95% CI for difference between means was 15.7%-22.2%; *P*<.001). Those who clicked on the fotonovela had a statistically greater percentage screened at the end of the QI project compared with those who did not click the fotonovela (95% CI for the difference between means was 3.2%-13.4%; *P*=.001). When only looking at patients who engaged in bidirectional texting (n=1026), those who did not click on the fotonovela had slightly higher screening rates than those who did click on the fotonovela (95% CI for difference of means was 0%-11.9%; *P*=.05).

### Barriers, Success Factors, and Suggestions for Increasing Screening Rates

In program week 2, the text messages queried the patients whether they had completed the FIT kit. If they had not completed the FIT kit or did not respond, they were asked about the barriers they were facing to complete the FIT kit. A total of 303 people responded to this question, 75 (24.7%) of whom replied “none” and 183 (60.4%) did not select a barrier from the list. Of those who chose a specific barrier from the list (n=45), the majority (19/45, 42%) said, “I feel fine, and I don’t have any symptoms.” The next highest selections were “I’m not sure why I need it” (n=10) and “I’m too busy right now” (n=10). Five people said that it was “embarrassing to do it and then mail it back,” and 1 person said, “I’m scared of the results.”

For those interviewed, the greatest motivator for returning the FIT kit was the patient navigator phone call, with the text message reminders and the fotonovela playing a smaller role (Table 4). The most common barrier reported by the 16 interviewees was simply forgetting to complete the FIT kit (9/16, 56%), followed by not wanting to take the time (7/16, 44%), followed by losing it or not remembering having received one (5/16, 31%).

Of the 16 interviewees, 15 (94%) cited mailing FIT kits to one’s home as a strong preference for going into the clinic, and they suggested that they continue doing this annually, with a more explicit offer via text message to request another kit to be mailed out if it was never received or lost. The simple instructions with pictures were specifically cited by 56% (9/16) of respondents as helpful. The 4 people who had trouble with instructions were all people who preferred the Spanish language, and 3 of them suggested more pictures and a video tutorial available via a weblink. Getting reminders via both phone and text message were both noted as helpful and unintrusive; none of the 16 people interviewed said they wanted to stop getting text messages or calls, and that having the option to text “stop” was sufficient. Patients had several suggestions for increasing motivation, including better advertising with a return date, adding an incentive in the form of a small gift card, or entry into a raffle. Finally, interviewees suggested offering more support, especially for first-time FIT kit users, such as the offer to walk through it at an upcoming clinic visit (Table 4).

## Discussion

### Screening Completion Among Usual Risk FQHC Patients

The QI project sought to use tailored texting with fotonovela comics to boost return rates for the FIT screening kit mailing campaign. Overall, the intervention group had a greater proportion of patients successfully screened at the end of the QI project compared with the usual care group, and this pattern was maintained for all demographic subgroups. This difference was driven by the significant increase in screening for the patients in the intervention group who engaged in texting, regardless of whether they clicked on the fotonovela. Women, Spanish speakers, and those with inconsistent screening histories (compared with very inconsistent or never screened histories) were more likely to be screened at the end of the QI project. The campaign was acceptable to the patients, although there were still many suggestions for further improvement. The effect seen here (7.2%) is stronger than what is known about the impact of text messages on CRC screening (0.6%-3.3% for CRC) [28] and similar to the effect of sharing a fotonovela booklet (7.1%) [29].

### Engaging FQHC Patients in CRC Screening

These results amplify the need to ensure that patients aged 61-75 years and those without insurance are not being left out of health promotion campaigns and a general need to continue to tailor materials and campaigns to maximize engagement and impact. There were clear differences in engagement by demographics; age and insurance status were related to both whether the patient would engage via bidirectional texting and whether they would click the fotonovela link. In addition, language, screening history, and SDOH needs were related to whether the patient would engage via bidirectional texting (although not in whether they clicked the link to the fotonovela).

In the study population, having half of the patients living in high or very high impact SDOH band areas drove the decisions for developing and tailoring the behavioral motivational messaging and the fotonovelas. Findings from previous research conducted with patients from this FQHC [5] provided information on known barriers to health behaviors that the team applied to frame and present information in culturally relevant formats. In the bidirectional texting program, 11.67% (303/2597) of the patients responded with a barrier to completing the FIT. These patient-reported barriers generally aligned with those noted in the literature: not knowing testing was necessary and lack of information [4,24,30,31], as anticipated in the automated responses to patient-reported barriers. Of note, a few test-specific barriers were noted, suggesting that materials accompanying the FIT addressed concerns about handling stool and other considerations that arise during the completion of a fecal test. Future work should investigate the timing of when it is most impactful to have the bidirectional texting program relative to when the FIT kits were mailed out.

Our results also showed the highest engagement via bidirectional texting for patients in the highest (greatest need) SDOH bands, indicating that these populations were open to communication. However, of the patients who did not engage in bidirectional texts, almost two-thirds were in the very high impact band. Of those who engaged but opted out, just more than half were in the very high impact band. It remains an important factor in future outreach strategies to tailor engaging and impactful ways of providing health services, especially when multiple social needs are unmet.

### Implications for Future QI

Although bidirectional texting appears beneficial, the platform and expertise it requires come at an additional cost for services that not all FQHCs may be able to afford; therefore, it would be useful to conduct a future campaign with the unidirectional texting that is more likely to be available to FQHCs and other clinics looking to boost CRC screening rates. A cost-benefit analysis of usual care compared with bidirectional texting with fotonovelas would also be useful to help determine which method to use in the long term. Similarly, although fotonovelas did not increase screening above and beyond bidirectional texting, it is possible that they would produce a boost beyond unidirectional texting, and this should be explored. Once created, fotonovelas do not incur substantial additional cost to use one-way texting blasts. Future exploration is needed to identify ways to encourage people to click on the fotonovela link.

The American Cancer Society recently updated the guidelines to reduce the recommended age to begin CRC screening from 50 to 45 years [32]. Health systems will need to explore ways to effectively reach out to younger patients who have not historically been screened. This may be more of a challenge, as previous research has found that patients are more likely to complete the FIT kit via mail if they have done once before [4], and younger patients might not be aware of the guidelines or feel that they are too young to worry about CRC.

With the COVID-19 pandemic resulting in decreased in-person clinical visits and pushing traditional interactions to telehealth, the FQHC is exploring how to best use text message and other phone-based promotions, communications, and programs to reach patients. Fotonovelas have historically been a print resource [33] but are less accessible to patients if they are only available in the clinics. The FQHC is exploring incorporating materials from this campaign to support patients in scheduling and preparing for colonoscopy, and other ways to use texting to reach patients for a broader range of clinical and social health needs over a longer term. It is also critical to continue to identify equity-centered methods that are useful and accessible for Hispanic and Latin American patients and other marginalized communities [34]. Newer technologies have the potential to significantly reduce the structural barriers to care.

### Limitations

The QI project tested whether the tailored text messages with fotonovela led to higher FIT kit return rates compared with usual care. However, when monitoring fotonovela link clicks, we found that those who clicked on the link did not have greater screening rates than those who merely engaged with texts without clicking. This finding could imply that the texting rather than the fotonovela was driving the increased screening in the intervention versus usual care groups or that the people likely to complete the FIT did so before receiving the fotonovela in week 3 of the intervention. In addition, it is possible that patients, despite not engaging, read the text messages, and those texts served as reminders for them to complete screening; the QI project could not attribute those screenings to the program components.

The interviews were a small, nonrandom convenience sample of clinical patients, with interviewees being, by definition, more engaged. Therefore, their feedback was viewed by the FQHC as potential ideas to explore, rather than definitive success factors and critical improvements. Similarly, the texted survey responses were a small nonrepresentative sample of responses, and although the data generally supported the findings from other methods in terms of barriers and enjoyment level of the fotonovela, it should not be considered definitive in nature, as selection bias was likely at play.

Owing to lags in data use agreement paperwork, the interviews were conducted over 2 months after the program ended and roughly 6 months after the FIT kits were originally mailed. This time lag may have affected the patients’ willingness to engage in interviews and their recall of the text messages and fotonovela.

The FQHC previously reported that 6.9% of the patients completing FIT had an abnormal result (ie, blood in the stool) [5]. In the current QI project, the usual care group FIT abnormal result rate (5.1%) compared with that of the intervention group (2.9%) suggests the importance of providing multimodal screening. This finding suggests that the usual care group had a higher baseline rate of abnormal results.

### Conclusions

Texting with automated conversational responses to those with a prior screening history appears to be valuable in increasing CRC screening. Patients were open to multiple contacts about their screening; a significant number of patients from all demographics engaged and returned FIT kits; and the vast majority of people who engaged with the campaign had positive or neutral responses, with very few indicating a negative impact. Intervention participants had moderately greater rates of returning FIT kits than those receiving usual care. Future work should tease out the differential impact of bidirectional texting versus unidirectional texting, and future campaigns could also attempt to address additional barriers raised by patients in the QI project. Finally, despite the success of this campaign, numerous patients remained unscreened, underscoring the need for continued education and multilevel interventions to reduce barriers to CRC screening.

## Appendix

Multimedia Appendix 1 Texting workflows.

## Abbreviations

CRC: colorectal cancer
FIT: fecal immunochemical test
FQHC: Federally Qualified Health Center
NLU: natural language understanding
QI: quality improvement
SDOH: Social Determinants of Health

## Data Availability

The data sets generated during or analyzed during this study are available from the corresponding author upon reasonable request.
