# Supplementary figures and images for: Using Text Messages and Fotonovelas to Increase Return of Home-Mailed Colorectal Cancer Screening Tests: Mixed Methods Evaluation
Source: JMIR Cancer. 2023 Mar 7;9:e39645. doi: 10.2196/39645 (PMC10131942; doi:10.2196/39645)

## Slide 1
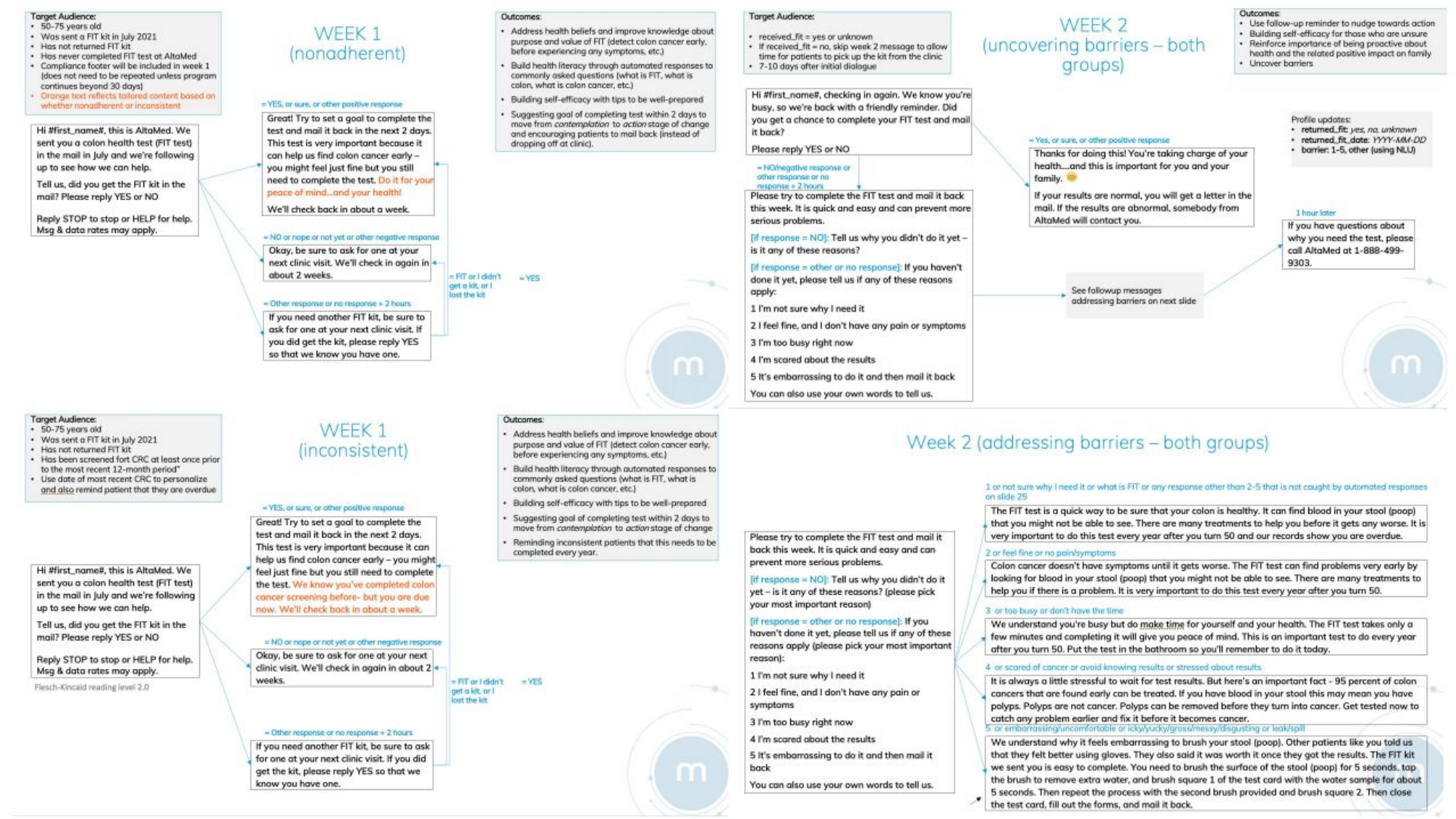

#

## Slide 2
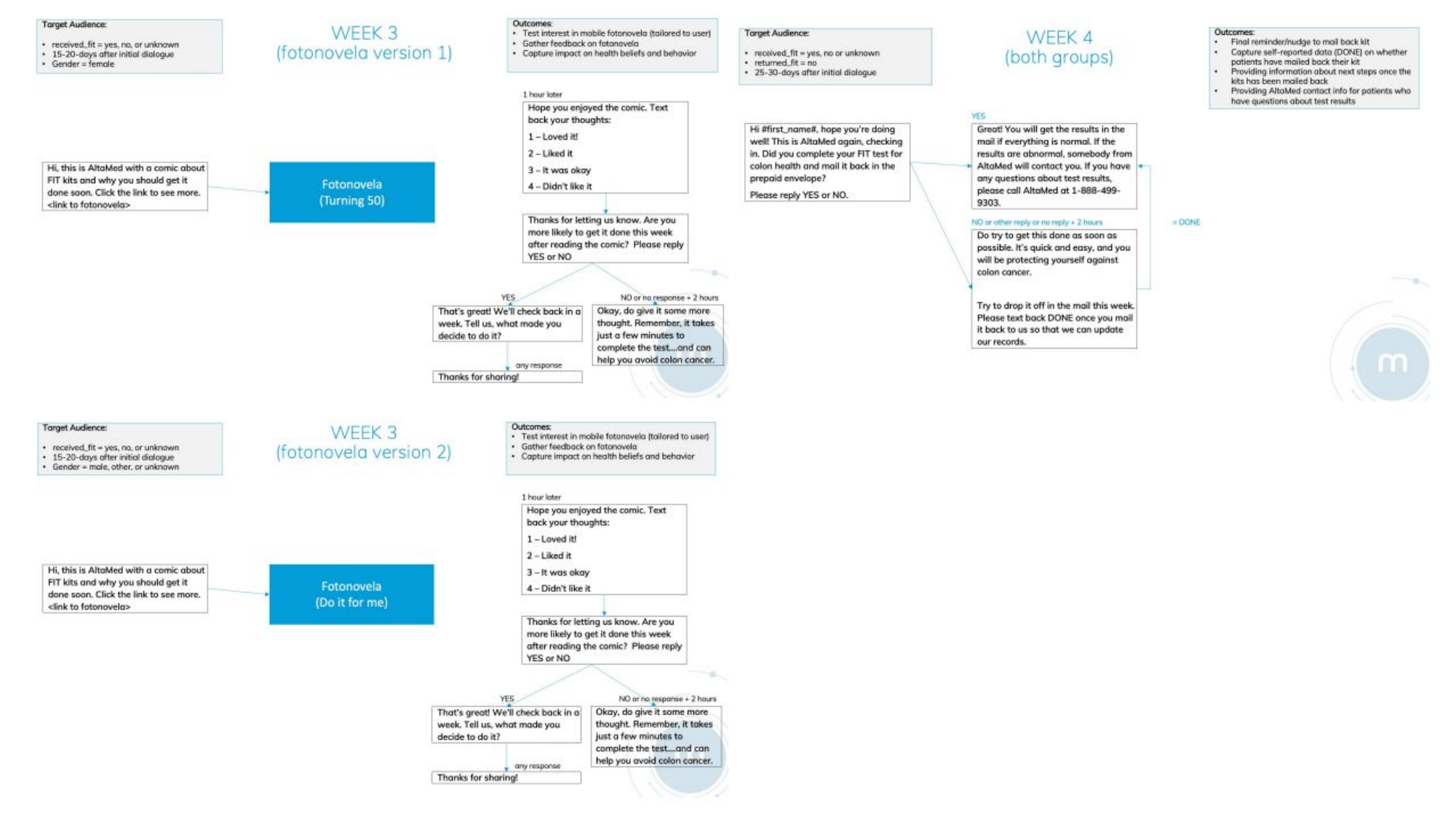

#

Supplement: Multimedia Appendix 1 [file cancer_v9i1e39645_app1.pptx]
